# Nurse Marion's Romance

**Published:** 1887-04-02

**Authors:** Kate Furley


### Nurse Marion's Romance.

By Kate Furley.

### Chapter II.—Marion's Home.

Marion—not Nurse Marion then, but Miss Jones, only child of Lieutenant Jones, R.N., retired and invalided—had even in those days been in unconscious training for her future vocation. The lieutenant was suffering from a complication of ailments, of which old age and bad temper were perhaps the most serious, though the latter was doubtless aggravated by the *sequelæ* of the wounds and fevers of his active days. Still, it was evident that "the old sea-dog," as Jack Holtayne contemptuously called him, might live for many years, if he had the continual care his daughter Marion lavished on him. If that were withdrawn, if he were left to such indifferent attention as his old servant might give him, a few months might terminate his career. Yet Jack Holtayne, who had fallen in love with Marion when on a brief visit to Saxford, the little seaport town where the lieutenant lived, and had easily won her unwary heart, being a handsome and pleasant-mannered youth of three-and-twenty, considered it very selfish in the old man to object to his daughter's leaving him to make her home a few thousand miles away. When he found that Marion did not consider her father's dislike to her immediate marriage absurd and unreasonable, he first called her weak, and then obstinate, by way of variation; and finally, when he found her firm in her resolve to remain at what she considered the post of duty, described her, too, as selfish and indifferent to him.

Marion tried to argue the point with him, tried to make him look at the matter from her point of view. It was useless, of course. It always is useless to argue with a man who thinks he is in love because he is in a temper, and measures his passion by his determination to have his own way. Marion's Jack left England with the firm conviction that he had been lavishing his affection on a girl who was wholly destitute of a heart, and would give up nothing for his sake. He was also convinced that his own heart was broken beyond possibility of repair. "But she shall never know how deep the wound is," he said to himself, with a savage delight in his own mock heroism; and, to achieve the desired concealment, he made love to an elderly, flirtatious damsel, who was going out to India in search of a husband, having failed in effecting a desirable capture of that nature at home, in spite of her long experience of the most approved style of going on the war-path, and her lavish expenditure on paint and feathers, and the English substitute for wampum beads.

This lady had no scruples about marrying young Holtayne, having long ago decided that the one duty she owed to herself and society was to establish herself in life as speedily and well as possible. Marry him she did, then; and made him as unhappy as he could deserve for a couple of years, at the end of which time a kind fever carried her off.

Meanwhile Marion, to whom the considerate Jack had sent a newspaper containing the announcement of his marriage, grew thin, and pale, and sad, till a new beauty, born of self-sacrifice, replaced on her countenance the bloom of girlhood that had waned too soon, and duty patiently done gave the needed strength for duty still remaining to do. Then, when patience had become peace, and endurance had attained contentment, the need for either ceased. Lieutenant Jones died, and his daughter was free. He had never shown any appreciation of the sacrifice she had made for him, had not spared her an atom of trouble or fatigue because of it; but a remark he made when he knew he was on his death-bed showed that he had felt it in some degree.

"You haven't had a very bright or easy life, Marion," he said; "and I know that you have given up what you thought was happiness for the sake of your grumpy old father. Perhaps the loss was less great than you fancied, and I hope and believe than some day you will receive tenfold what you lost by remaining with me. God is a generous giver, but He takes His own time to give."

To Marion it seemed that these words more than atoned for all past loss; but friends who held more practical, perhaps more cynical views of life, said that since the lieutenant knew he must leave his daughter penniless he had no right to prevent her accepting a home of her own when it was offered to her. His income had been but a small one, and it had died with him; Marion must now go out in the world and work for herself. The usually insoluble problem of what a woman is fit for who has had no special training for any calling, was more easily decided in her case than in most. Her long attendance on a most exacting invalid had proved her to be of the material from which a good nurse may be made, and it was felt that she had done wisely when she elected to go to a hospital and study nursing as a profession.

Since then her life had gone on in a calm and busy round. If the past was not forgotten it was put aside, and neither hope nor memory was allowed to interfere with the work she had to do. She had heard nothing of Jack Holtayne since he sent her the announcement of his marriage, and it had never seemed possible to her that any new love should efface the old. In early days, when she had been one of the prettiest probationers at the Liverpool Hospital, an occasional student had expressed his admiration for her, but she had felt no difficulty in putting aside such demonstrations, and it had never occurred to her that it was even possible that she should accept their love. By the time she came to the hospital of Saint Lazarus, to take charge of Dr. Brandreth's ward, she was quite convinced that she was too old and faded for any man to think her worth looking at, and surprise was stronger than any other conscious emotion when it became evident to her that the doctor did not share her opinion.

"But why should the matter disturb me so much?" she asked herself. "Is it because he is an older man than any who has ever liked me before? It does seem hard to give him pain; I like and respect him so much, and I do not see how I can remain in his ward after—What a pity it is! And I shall have to go to some hospital, where there will be no other Nurse Jones, and I shall have to return to that not very euphonious title."

For our heroine was by no means painfully free from vanity, and had not been sorry that there should be another, senior Nurse Jones at St. Lazarus', for which reason she was known by her Christian name, which she much preferred.

Dr. Brandreth kept his promise not to do anything to influence her treatment of his suit. Marion was not often alone with him during the next two days, and when they did meet out of earshot of others he was careful to speak of nothing but the work they shared. On the third day, however, he broke down for a moment. The mysterious patient, Mr. Hatton, had arrived, and had been borne to the bed prepared for him in the little private ward, which Nurse Marion had somehow by her dainty touches made less bare than it is the wont of hospital rooms to be. Dr. Brandreth was superintending the arrival, when he was surprised to see Nurse Marion, who met the patient in the ward, turn pale, and after giving a few directions in an almost inaudible voice, leave the apartment.

As soon as he could he followed her, and found her in the corridor, leaning for support against the wall.

"What is the matter?" he asked. "Are you ill?"

"No, I am not ill," she answered. "I shall be all right soon. It was the surprise that overcame me for a moment. That new patient is—Jack Holtayne, of whom I told you the other day."

*(To be continued.)*

